# The Influence of School Policies on Smoking Prevalence Among Students in Grades 5-9, Canada, 2004-2005

**Published:** 2010-10-15

**Authors:** Chris Y. Lovato, Allison W. Pullman, Peter Halpin, Cornelia Zeisser, Candace I.J Nykiforuk, Frankie Best, Alan Diener, Steve Manske

**Affiliations:** School of Population and Public Health, University of British Columbia; University of British Columbia, Vancouver, British Columbia, Canada; University of British Columbia, Vancouver, British Columbia, Canada; University of British Columbia, Vancouver, British Columbia, Canada; University of Alberta, Edmonton, Alberta, Canada; British Columbia Ministry of Healthy Living and Sport, Victoria, British Columbia, Canada; Public Health Agency of Canada, Ottawa, Ontario, Canada; Propel Centre for Population Health Impact, University of Waterloo, Waterloo, Ontario, Canada

## Abstract

**Introduction:**

School characteristics may account for some of the variation in smoking prevalence among schools. The purpose of this study was to investigate the relationships between characteristics of school tobacco policies and school smoking prevalence. We also examined the relationship between these characteristics and individual smoking status.

**Methods:**

Tobacco policy data were collected from schools in 10 Canadian provinces during the 2004-2005 school year. Written tobacco policies were collected from each school to examine policy intent, and school administrators were surveyed to assess policy enforcement. Students in grades 5 through 9 completed the Youth Smoking Survey to assess smoking behaviors and attitudes. We used negative binomial regression and multilevel logistic regression to predict the influence of school policies on smoking behavior at the school and student levels.

**Results:**

School policies that explicitly stated purpose and goals predicted lower prevalence of smoking at the school and individual levels. Policies that prohibited smoking on school grounds at all times predicted lower smoking prevalence at the school level but not at the individual level.

**Conclusions:**

For maximum effectiveness, school smoking policies should clearly state a purpose and goals and should emphasize smoking prohibition. These policies can help reduce smoking prevalence among youths and are part of a comprehensive school approach to tobacco control.

## Introduction

Environmental factors influence smoking behaviors (1,2), and the school environment is an important setting for substance use prevention efforts (3). Schools are places where social behaviors are modeled and reinforced, and they are identified in ecologic models as an influence proximal to behavior (4-6). School-based strategies are a key element of tobacco control among young people because school environments are established systems where smoking behaviors can be targeted (4). Policies are necessary for comprehensive school-based tobacco control, but little is known about the characteristics of school policies and their relationship to tobacco use.  Additionally, smoking rates vary among schools, even after controlling for individual characteristics, suggesting that school context contributes to student smoking (7,8).

Tobacco control policies targeted at the population level have been a successful public health strategy, but school smoking policies have had mixed effects on individual behavior (4,7-17). Several studies indicate that schools with tobacco control policies, such as smoking bans, have lower smoking prevalence (14-16). Smoking behavior is related to policy strength (13), and school tobacco policies are effective when strongly enforced (7,10,16-18).

A school smoking policy is considered to be strong if it was developed with input from students and is comprehensive, consistently enforced, and addresses prevention education and cessation strategies (7,19). A review examining the elements of tobacco control policies found that only bans and policy enforcement elements deterred smoking (20), and another study found that school smoking policies are ineffective at reducing smoking uptake among adolescents (12). By the time children reach secondary school, policy enforcement appears to lose its protective effect, and students' perceptions of strong rules can indicate higher smoking prevalence (4).

Both policy content and implementation need to be considered when predicting smoking behavior (7). Measuring school policy intent and enforcement will further our understanding of how policies may affect smoking behavior.

Both individual and community factors influence smoking behaviors, and the prevalence of smoking varies from school to school. This variation suggests that an ecologic analysis is necessary to understand student smoking behaviors. Yet most research on school smoking policies has focused on examining the relationship of policies to individual smoking status. Although we acknowledge that the purpose of school smoking policies is to influence individual smoking behavior, school policies are primarily intended to focus on the environment by encouraging and reinforcing nonsmoking norms within the school setting.

The purpose of this study was to examine how policy characteristics are associated with school smoking prevalence. We conducted a secondary analysis to examine how these characteristics influence the smoking status of individual students.

## Methods

### Participants

A total of 281 elementary and secondary schools in 10 Canadian provinces were recruited (55% response rate) as part of the 2004-2005 Youth Smoking Survey (YSS) (21) conducted by the University of Waterloo. The YSS, a biennial survey sponsored by Health Canada, provides national and provincial data on tobacco attitudes and behaviors among children and adolescents. Schools in the Yukon, Nunavut, and Northwest Territories were excluded from the sample. The sampling was conducted in 2 stages. First, school boards were selected within each province, and second, schools were sampled from the selected boards. The sample featured 3 levels of stratification: province, health region smoking rate, and grade level. School boards were randomly selected within the stratum, and the probability of inclusion was weighted according to the number of students in the board. Both public and private schools were included in the sample. In each school, students in grades 5 through 9 were eligible to complete a survey about their smoking attitudes and behaviors (Table 1).

In conjunction with the YSS, school administrators were asked to provide all written documentation pertaining to the school's smoking policy at the time of data collection. At each school, an administrator who was knowledgeable about the smoking policy was interviewed to assess enforcement. This study was approved by the University of British Columbia behavioral research ethics board.

### Data sources and measures

**Student survey**

Student smoking behaviors were assessed by the YSS (21). The YSS uses the tobacco module of Canada's School Health Action, Planning and Evaluation System, which has been established as a valid and reliable measure (22). The instrument is a machine-readable, paper-and-pencil questionnaire completed by the student in the classroom. National data on smoking prevalence, tobacco purchasing behaviors, tobacco marketing, school smoking policies, and the prevalence of alcohol and drug use were collected. Active parental permission and student consent were required for participation. Of the 51,285 eligible students, 29,553 returned the questionnaire (58% response rate).

We measured smoking status as a binary outcome at the student level (1 = smoker, 0 = nonsmoker). A smoker was defined as having smoked at least 100 cigarettes in his or her lifetime and also having smoked, even just a puff, in the last 30 days. At the school level, smoking prevalence was calculated as a continuous variable by dividing the number of respondents identified as smokers by the total number of respondents.

**Written policy (policy intent)**

To assess policy intent, we examined the school's written smoking policy. A policy could either be the school's own policy or, in cases where schools did not develop their own policy, the school board policy. Some schools had both their own policy and a board policy, in which case the school policy was used. We omitted from analysis schools that did not have their own policy or a board policy.

To quantify policy intent, we used a coding scheme adapted from a validated school policy rubric (17,19,23); higher scores reflect stronger policies (Table 2). We modified this rubric to reflect the Canadian context and recent theoretical findings (18,24,25). The substantive aspect of construct validity for the coding protocol was acceptable (22). Where appropriate, internal consistency (Cronbach α) is reported (Table 2). Written policies from each school were independently coded by 2 trained staff members. All coding discrepancies were documented and discussed until consensus was reached. Interrater reliability was high, with an average agreement of 97%.

**Administrator survey (policy enforcement)**

To assess policy implementation, we developed a survey that incorporated school health questionnaires (23,24) and guidelines from published policy research (18,19). The survey was pilot-tested with 3 school administrators (not included in our sample) before it was finalized. We interviewed the school principal, vice principal, counselor, or teacher most knowledgeable about the school's tobacco policy. Interviewees answered questions about who was involved in policy development, how students were informed, and the nature of enforcement. From their responses, we used 3 items that describe the schools' enforcement of their tobacco policy (Table 3).

**Grade**

Two comparable variables were used to examine student or school grade. For the school-level analysis, the highest grade at the school (eg, 12) was used to indicate the potential influence of older students. For the student-level analysis, the grade of the respondent was used as a control variable. For this level of analysis, highest grade at the school was not a significant covariate after students' grades were included; thus, we omitted this variable from the analysis.

### Analysis

For our full models, we tested the relationship between the score for each policy variable and the school smoking prevalence or student smoking status. Using type 3 hypothesis testing of the variables included in these models (25), all variables with a regression coefficient that met the *P* < .10 level of significance were further tested in a reduced model controlling for age and sex. Regression coefficients and their associated *P* values are not reported. Final significance was set at *P* < .05.

Negative binomial regression analysis was used to examine the relationship between school policy characteristics and school smoking prevalence. This approach was selected to account for overdispersion of smoking prevalence (mean, 1.53%; standard deviation, 3.08%). The distribution of smoking prevalence was nonnormal and was too skewed to allow traditional methods of transforming the data for linear modeling. This nonnormal distribution was due to a high number of schools at both extremes of smoking prevalence; 61% of schools had no identified smokers. For the purposes of the negative binomial regression, smoking prevalence was represented as a count or discrete variable instead of a continuous variable. The fit of the negative binomial distribution was adequate for both the full and reduced models (full model: χ^2^ / *df* = 0.8990; reduced model: χ^2^ / *df* = 0.8554).

Multilevel logistic regression analysis was used to determine the relationship between school policy characteristics and individual smoking status. Pearson χ^2^ analysis indicated that the overall fit of the logistic model was adequate for both the full and reduced models (full model: χ^2^ / *df* = 0.9929; reduced model: χ^2^ / *df =* 0.9795).

For all variables included in the models (Tables 2 and 3), higher values represented a stronger policy. Variables with 3 or fewer levels were coded as ordinal indicators. Variables with more than 3 levels were treated as continuous. Survey weights were applied to the student data to derive population estimates and to adjust for sampling methods. Statistical analyses were conducted using SAS version 9.1.3 (SAS Institute, Inc, Cary, North Carolina).

## Results

Of the 281 schools recruited, complete data were available for 272 schools and 27,892 students in grades 5 through 9. Students were approximately evenly distributed by sex (girls, 53%) and grade level.

The overall smoking prevalence was 1.5%; there was no significant difference in smoking status by sex. The grade configuration of each school (by highest grade at school) is reported in Table 4.

Eight percent of schools had no written tobacco policy. The mean smoking prevalence was highest for schools with only a school-developed policy (2.6%), followed by schools with their own policy and a district policy in place (1.6%), schools with only a district policy in place, (1.2%), and schools with no policy (0.7%).

### School smoking prevalence

Predictors of school smoking prevalence that were retained from the full model were the highest grade level at the school, 3 policy intent variables (purpose and goals, smoking prohibition, and assistance overcoming tobacco addictions), and 1 enforcement variable, the presence of an enforcement officer (Table 5). The highest grade level at a school predicted school smoking prevalence. Written school policies with a stated purpose and goals and strong prohibition predicted lower school smoking prevalence. Written school policies that mandated cessation programs predicted higher school smoking prevalence. Having a person designated as responsible for policy enforcement was not a significant predictor of school smoking prevalence.

### Student smoking status

Predictors of student smoking status that were retained from the full model were the student's grade, 3 policy intent variables (purpose and goals, smoking prohibition, and assistance overcoming tobacco addictions), and 1 enforcement variable, the presence of an enforcement officer (Table 6). A student in a higher grade was more likely to smoke than a student in a lower grade. Written policies with a statement of purpose and goals decreased the likelihood that a student was a smoker. A student was more likely to smoke if he or she attended a school that mandated cessation programs. Prohibition of smoking and designation of a person responsible for enforcement of the tobacco policy were not significant predictors of student smoking behavior, but both were in the expected direction.

## Discussion

We found that school smoking policies can influence individual smoking status at both the school and individual levels, which is consistent with previous studies (7,14,18). Policies that address purpose and goals and that prohibit smoking by all people and at all times are associated with lower school smoking prevalence. School bans have previously been reported as effective (8,14-16); thus, we conclude that prohibiting smoking by all people and at all times is a key policy message.

Policies that included a clearly stated purpose and goals predicted less smoking at both the individual and school levels. A clearly stated rationale may suggest a more established tobacco control strategy or a stronger commitment by school administrators to address smoking issues. Policy guidelines indicate that purpose and goals are key components of a good policy (19,23,26,27).

Schools with written policies that mandated cessation programs had higher smoking rates at both school and individual levels. Schools with many students and staff who smoke likely would have had more reason to develop and mandate tobacco cessation programs. The cross-sectional nature of this research does not allow us to address this question.

Most (92%) schools in this study had a written school or board tobacco policy in place. This finding is encouraging and suggests that schools are taking action to reduce and prevent student tobacco use. However, the policies were generally weak. In particular, scores for developing, overseeing, and communicating policy and strength of enforcement were very low, which may explain their lack of statistical significance. Also, many of the written policies, particularly in elementary schools, were simple excerpts from a student handbook and not fully developed. Anecdotally, many school personnel commented that they did not feel that a policy was necessary for an elementary school.

Older students were more likely to smoke than were younger students, and smoking rates among students in grades 5 through 9 were higher in schools with older students (up to grade 12) than in schools with younger students. Having older students at a school appears to influence the smoking behavior of younger students, which confirms similar findings (28,29).

In recent years, schools have been encouraged to provide tobacco use prevention education as part of an effective strategy. The effectiveness of these programs is mixed (12,30-32). In our study, policies that mandated tobacco use prevention education were not associated with school or student smoking rates. In other research we have conducted, schools with a strong focus on prevention education had lower smoking rates (33).

We found that many elements of school tobacco policies were not associated with smoking behaviors. Most policy characteristics alone likely account for only small variations in smoking. Many factors work together to influence smoking, including individual, school, and community factors that were not measured in this study. A study by Murnaghan et al (11) revealed that a combination of tobacco control programs and school tobacco policies were protective of occasional smoking only among students who perceived clear smoking rules. It is possible that school policies are more effective for certain students and when combined with other tobacco control efforts. No single factor accounts for all variance in student behavior, and some factors (eg, programs and policies) may have synergistic effects. Canada's Joint Consortium on School Health supports a school self-assessment tool that focuses on a tobacco policy as well as prevention and cessation programming (34).

Many studies have used multilevel analysis to address factors related to smoking, but this approach makes it difficult to "disentangle effects with observational data sets" (5,35). We found that results from the individual-level analysis were similar but not identical to school-level findings, suggesting that separate analyses should be considered. Further work is needed to guide researchers in this area.

This study has limitations that should be considered. First, students were in grades 5 through 9, where smoking rates tend to be lower than among older students. We coordinated this study with a national survey of youth focused on this age group. The survey has been recently expanded to include older students. Second, the coding rubrics in this study need to be further tested for reliability and validity. Policy scores derived from our coding scheme were restricted in range, particularly for certain items, which may have limited our ability to detect any relationship with smoking. Finally, data in this study are cross-sectional. Longitudinal analyses examining smoking and the school environment are needed to better understand the effects of school context on smoking behavior.

Despite these limitations, this study contributes to research on school tobacco policies by focusing on the school outcomes, the level to which policies are directed. On the basis of our results and the existing research, we conclude that school smoking policies can contribute to reducing youth smoking as part of a comprehensive school approach to tobacco control. To maximize impact, policies should describe their purpose and goals and should emphasize smoking prohibition.

## Figures and Tables

**Table 1:** Sampling Framework, Youth Smoking Survey, Canada, 2004-2005

Province	Selected Boards by Stratum			Selected Schools by Stratum	

	High^a^	Low^b^	Other^c^	Junior^d^	Senior^e^
Newfoundland and Labrador	0	0	4	12	12
Nova Scotia	0	0	5	11	13
Prince Edward Island	0	0	2	14	10
New Brunswick	2	2	0	8	12
Quebec	4	5	1	24	12
Ontario	5	6	1	11	30
Manitoba	3	3	2	11	17
Saskatchewan	2	3	1	3	19
Alberta	5	2	1	10	20
British Columbia	4	4	1	16	16

a School boards in a health region with a smoking rate at the median or higher.

b School boards in a health region with a smoking rate lower than the median smoking rate.

c All school boards in Newfoundland and Labrador, Nova Scotia, and Prince Edward Island were selected. This stratum also includes private, French language, and First Nation school boards for provinces in which these are administratively separate from public boards.

d Schools with students in grades 5, 6, 5-6, and 6-7.

e Schools with students in grades 5-8, 5-9, 6-8, 6-9, 7, 7-8, 7-9, 8, and 9.

**Table 2:** Sample Items Used to Code Policy Intent Variables, Youth Smoking Survey, Canada, 2004-2005

**Variable**	Sample Items	Scoring Range	Cronbach α^a^
Developing, overseeing, and communicating policy	Is the tobacco policy written? Who should be involved in the development of tobacco policy? How should the policy be communicated to students, staff, and parents? Does the tobacco policy outline consequences of students, staff, and/or parents breaking the rules?	0-14	.67
Purpose and goals	Are the intent and rationale of the tobacco policy outlined?	0-1	NA
Smoking prohibition	Does the policy prohibit smoking of tobacco by students?	0-1	NA
Possession prohibition	Does the policy prohibit possession of tobacco by students?	0-1	NA
Strength of enforcement	Does the policy specify how often specific punishments, referrals, and mandatory programs are administered when students violate the tobacco policy?	0-9	.67
Characteristics of enforcement	Does the tobacco policy specify that sanctions should get stronger with repeat offenses? Is there a person who is designated as primarily responsible for enforcing policy?	0-2	.42
Tobacco use prevention education	Does the tobacco policy mandate that all students receive instruction to avoid tobacco use?	0-1	NA
Assistance to overcome tobacco addictions	Does the tobacco policy specify the availability of cessation programs for students and/or staff?	0-1	NA

Abbreviation: NA, not applicable.

a Computed for school-level data; student-level data showed comparable values.

**Table 3:** Items Used to Code Policy Enforcement Variables, Youth Smoking Survey, Canada, 2004-2005

**Variable**	Description	Scoring Range	Cronbach α
Enforcement officer	Does the school designate a person who has primary responsibility for enforcement of tobacco use policy?	0-1	NA
Consistency of tobacco policy enforcement (students)	How consistently is tobacco policy enforced with students (never to always)?	0-3	NA
Consistency of tobacco policy enforcement (other)	How consistently is tobacco policy enforced with teachers or staff, parents, and school visitors?	0-9	.92

Abbreviation: NA, not applicable.

**Table 4:** Highest Grade in Participating Schools, Youth Smoking Survey, Canada, 2004-2005

**Highest Grade**	No. of Schools (%), N = 272
5	17 (6)
6	81 (30)
7	18 (7)
8	54 (20)
9	21 (8)
10	4 (1)
11	5 (2)
12	72 (26)

**Table 5:** Predictors of School Smoking Prevalence for Students in Grades 5-9, Youth Smoking Survey, Administrator Survey, and Collected School Written Policies, Canada, 2004-2005

**Model/Policy Variable**	Relative Risk (95% CI)	*P* Value^a^
**Full Model**		
Highest grade present at school	1.66 (1.47-1.88)	<.001
**Policy intent variables**		
Developing, overseeing, and communicating policy	1.02 (0.80-1.28)	.93
Purpose and goals	0.64 (0.36-1.18)	.15
Smoking prohibition	0.44 (0.20-0.98)	.04
Possession prohibition	0.61 (0.34-1.12)	.10
Strength of enforcement	1.02 (0.88-1.20)	.79
Characteristics of enforcement	1.06 (0.69-1.61)	.80
Tobacco use prevention education	0.65 (0.23-1.78)	.40
Assistance to overcome tobacco addictions	2.33 (0.98-6.00)	.06
**Policy enforcement variables**		
Enforcement officer	0.65 (0.39-1.08)	.09
Consistency of enforcement (students)	1.03 (0.55-1.87)	.92
Consistency of enforcement (other)	0.97 (0.80-1.17)	.69
**Reduced Model**		
Highest grade present at school	1.65 (1.47-1.87)	<.001
**Policy intent variables**		
Purpose and goals	0.57 (0.34-0.99)	.04
Smoking prohibition	0.43 (0.20-0.97)	.04
Assistance to overcome tobacco addictions	2.15 (1.10-4.37)	.03
**Policy enforcement variables**		
Enforcement officer	0.65 (0.39-1.06)	.08

Abbreviation: CI, confidence interval.

a Calculated by using χ^2^ test.

**Table 6:** Predictors of Smoker Status a for Students in Grades 5-9, Youth Smoking Survey, Administrator Survey, and Collected School Written Policies, Canada, 2004-2005

**Model/Policy Variable**	Odds Ratio (95% CI)	*P* Value^b^
**Full Model**		
Student's grade	2.81 (1.93-4.09)	<.001
Male sex	0.82 (0.54-1.23)	.33
**Policy intent variables**		
Developing, overseeing, and communicating policy	0.79 (0.57-1.09)	.14
Purpose and goals	0.40 (0.14-1.14)	.08
Smoking prohibition	0.54 (0.23-1.26)	.15
Possession prohibition	0.97 (0.48-1.96)	.95
Strength of enforcement	0.97 (0.83-1.11)	.67
Characteristics of enforcement	1.31 (0.74-2.38)	.35
Tobacco use prevention education	0.69 (0.18-2.66)	.29
Assistance to overcome tobacco addictions	2.69 (1.34-5.42)	.005
**Policy enforcement variables**		
Enforcement officer	0.60 (0.36-0.99)	.05
Consistency of enforcement (students)	1.63 (0.55-4.82)	.38
Consistency of enforcement (other)	0.91 (0.64-1.30)	.62
**Reduced Model**		
Student's grade	2.81 (1.92-4.12)	<.001
**Policy intent variables**		
Purpose and goals	0.38 (0.15-0.95)	.04
Smoking prohibition	0.53 (0.22-1.26)	.15
Assistance to overcome tobacco addictions	2.23 (1.12-4.45)	.02
**Policy enforcement variables**		
Enforcement officer	0.62 (0.37-1.04)	.07

Abbreviation: CI, confidence interval.

a Smoker status was defined as having smoked at least 100 cigarettes in his or her lifetime and having smoked, even just a puff, in the last 30 days.

b Calculated by using χ^2^ test.
